# Chem4Energy: a consortium of the Royal Society Africa Capacity-Building Initiative

**DOI:** 10.1098/rsfs.2024.0001

**Published:** 2024-08-09

**Authors:** Marietjie J. Ungerer, Evans Adei, Theopolina Amakali, Cecil H. Botchway, Likius S. Daniel, James Darkwa, Nelson Y. Dzade, Foster Mbaiwa, Mary Mensah, Maipelo Nyepetsi, Banothile Makhubela, Claire E. Mitchell, Oluwasegun Emmanuel Olaoye, Olayinka A. Oyetunji, Meenakshisundaram Sankar, Fortunate P. Sejie, Jacobina Sheehama, Richard Tia, Veikko Uahengo, Aleksandar Živković, Nora H. De Leeuw

**Affiliations:** ^1^ School of Chemistry, Cardiff University, Main Building, Park Place, Cardiff CF10 3AT, UK; ^2^ School of Chemistry, University of Leeds, Leeds LS2 9JT, UK; ^3^ Department of Chemistry, Kwame Nkrumah University of Science and Technology, Kumasi, Ghana; ^4^ Department of Physics, Chemistry and Material Science, University of Namibia, Windhoek, Namibia; ^5^ Department of Chemistry, University of Botswana, Gaborone, Botswana; ^6^ Department of Energy and Mineral Engineering, Pennsylvania State University, University Park, PA, USA; ^7^ Department of Chemical and Forensic Sciences, Botswana International University of Science and Technology, Palapye, Botswana; ^8^ Department of Chemistry, University of Johannesburg, Johannesburg, South Africa; ^9^ Department of Earth Sciences, Utrecht University, 8a Princetonlaan, 3584 CB Utrecht, The Netherlands

**Keywords:** collaboration, sustainable energy, capacity building

## Abstract

The Africa Capacity-Building Initiative is a Royal Society programme funded by the former UK Department for International Development to develop collaborative research between scientists in sub-Saharan Africa and the UK. Initially, four institutions were involved in the Chem4Energy consortium: Cardiff University in the UK and three African partners, the Kwame Nkrumah University of Science and Technology, Ghana, the University of Namibia and the University of Botswana, soon also including the Botswana International University of Science and Technology. The Chem4Energy research programme focused on ‘New materials for a sustainable energy future: linking computation with experiment’, aiming to deploy the synergy between state-of-the-art computational and experimental techniques to design and optimize new catalysts and semiconductor materials for renewable energy applications, based on materials that are abundant and readily available in African countries. The Chem4Energy consortium has achieved ambitious research goals, graduated seven PhD students and delivered a high-quality cross-disciplinary training programme in materials science and simulation techniques relevant to renewable energy applications. Since 2021, the extended consortium, including North-West University and the Centre for High-Performance Computing in South Africa, has remained active through an annual Chem4Energy conference series, with the sixth meeting taking place in Namibia in April 2025.

## 1. Introduction

The Chem4Energy consortium was awarded a grant by the Royal Society in 2015 for their project entitled ‘New materials for a sustainable energy future: linking computation with experiment’ under the Africa Capacity-Building Initiative (ACBI), funded by the UK Department for International Development (now the Foreign, Commonwealth and Development Office). The focus of the Chem4Energy research capacity-building activities—and the ACBI programme more generally—was to train a cohort of PhD students and associated researchers in-country, as part of an equal partnership between the UK and African institutions. This approach avoided the need to bring early career researchers out of their country with the very real risk that they would not return, adding to the unsustainable brain-drain suffered by many countries in the Global South.

The diverse skills and research interests of the partners in the consortium provided an excellent opportunity to combine computational work with complementary experimental research. The research programme focused on two themes in the areas of sustainable energy:

— Development of benign catalysts for the conversion of biomass from waste to fuels and chemicals.
— Development of novel efficient solar cell materials.

The Chem4Energy consortium was built on the existing partnership between De Leeuw, then at University College London, but who moved to Cardiff University (CU) in 2015, and Adei and Tia at the Kwame Nkrumah University of Science and Technology (KNUST) in Ghana, who together had received an earlier grant from the Royal Society using Leverhulme Trust funding to form a research and training collaboration in computational energy materials science. Oyetunji from the University of Botswana (UB) and Uahengo from the University of Namibia (UNAM) provided complementary experimental expertise. These four institutions (CU, KNUST, UB and UNAM) formed the original Chem4Energy consortium under the ACBI programme. However, early on colleagues with computer modelling experience from the relatively new Botswana International University of Science and Technology (BIUST) joined the consortium, which has also benefitted hugely from the participation of the Centre for High-Performance Computing (CHPC) in South Africa, who provided internships for the PhD students, computer clusters and training for the participating institutions and access to CHPC resources to the Chem4Energy researchers.

The chosen research project aimed to harness the power of predictive computer modelling in a synergistic programme with experiment to design and develop new materials for the production of sustainable (solar) fuels or chemical feedstock. Finite fossil fuel reserves and the threat of climate change from rising carbon dioxide levels have put renewable energy firmly on the global agenda, which was, therefore, an excellent topic for scientific investigation. Our collaborative research programme aimed to train a cadre of African scientists in the use of state-of-the-art computer modelling and advanced experimental techniques to design, develop and optimize new materials for solar cells and energy storage devices and the production of low-carbon sustainable fuels or chemical feedstock. The project focused on three promising classes of materials:

- homogeneous transition metal catalysts for hydrogenation and functionalization of biomass components, e.g. lignin, for applications as biofuels, in energy storage materials and as a source of chemical feedstock;
- copper/zinc-based thin films for solar cells; and
- microporous materials for the sorption and conversion of biomass, where we aimed to identify optimum architectures for reactants and products and design active sites within the porous structures to facilitate catalytic conversion to fuels or chemicals.

The unique strength of the Chem4Energy consortium was the synergy between computation and experiment, fully integrating state-of-the-art computational techniques with advanced materials synthesis and characterization. Furthermore, we focused in the research on developing local sustainable energy sources, deploying materials that are readily available in the African partner countries, e.g. copper deposits in Namibia, non-edible fruits and tallow waste from the meat industry in Botswana and zeolites made from large clay deposits in Ghana.

## 2. The Chem4Energy research and training programme

The Chem4Energy UK–Africa collaborative research and training initiative was set up to achieve a truly complementary programme of computational and experimental research—initially with two experimental and two computational partner groups—to benefit from the synergy between computation and experiment in obtaining the research outcomes. A crucial part of this consortium is the complementarity of the research being carried out by each partner: UB partners have reported the synthesis and testing of catalysts for successful partial hydrogenation of oils from non-edible plants. Alongside this experimental work, KNUST partners have identified the most promising catalysts for these processes via the calculation of reaction mechanisms. The UNAM partners have worked on the development of copper and zinc oxide (ZnO) semiconductors for solar applications, with band gaps in very specific regions, whereas the CU team have provided modelling expertise, both predicting and explaining the properties of the newly developed solar materials.

The students were selected in a highly competitive process, based on the strength and relevance of their CV and performance at interview. The selection process went through multiple levels in the departments before the final approval authority made a decision. It was decided to have at least two students for each participating institution and the students were supervised by at least two consortium team members in their own institution, as per the regulations of the university where they were registered. In addition, the students were mentored informally by other consortium members at the biannual in-person consortium meetings and through virtual meetings.

The training programme was designed with travel in mind, not only to maximize the exposure of students to different research environments but also to foster new collaborations spanning continents and research fields. Various workshops were hosted and attended, with topics ranging from crystallography and the Cambridge Crystallographic Data Centre (2015), using modelling codes including Gaussian [1] and Vienna Ab initio Simulation Package (VASP) [2–5], molecular dynamics (MD) simulations and the use of the DL_POLY program [6] (2016), attending the Royal Society of Chemistry ‘Catalysis for fuels’ Faraday Discussion (2017), public outreach of science and developing and protecting Intellectual Property at CU (2018), participating in the 7th Federation of African Societies of Chemistry (FASC) meeting in Botswana (2019), to transferrable skills in CV writing, the interview process, writing research papers, ethics in research, plagiarism and referencing, public engagement, training in knowledge transfer and exploitation, project management and entrepreneurship.

The PhD students followed taught courses during their PhD programmes, as required by their individual home institutions. They were also trained in specific techniques relevant to their research by their project supervisors in CU, KNUST, UNAM and UB. In addition, the students, including wider groups of interested staff and graduates, have benefitted from an extensive bespoke programme of cohort-wide training courses, including transferable and generic skills development, as well as more generic technical training, e.g. data analysis, materials characterization and digital skills. These training activities rotated between three African partner institutions to maximize participation. The partner teams plus a range of external specialists who delivered this training programme have thus enabled the PhD students and wider participants to benefit from the following research and training activities.

- Technical training
- Transferable and generic skills development
- Accredited project management
- Entrepreneurship and exploitation
- Knowledge exchange and public outreach
- Responsible innovation
- Employability
- Collaborative research in renewable energy topics.

## 3. Development of a research capacity-building plan

In the planning stages of such a consortium, it was important to build in flexibility and adaptability, as initial plans might have been needed to be adapted during the course of a 5- or 6-year programme. Keeping in mind that this programme was meant to build research capacity in sub-Saharan African institutions, we had to consider the training and resources required by our students to become future research leaders.

The main aim was to create a long-term partnership between the UK and African partner institutions, training and supporting PhD candidates in each institution on research pertaining to the theme ‘New materials for a sustainable energy future: linking computation with experiment’, but also building the research capacity of the wider African teams, so that their research would be sustainable and capable of continuing beyond the funded period. The first aim was met by creating a scientific community within this consortium and building collaborations between the different groups, focusing in particular on building sustainable and complementary South–South collaborations, and including partners from South Africa, who could provide further and continuing local support to the African partners. It was part of the bigger picture to encourage women to join the consortium and succeed in a scientific careers. Second, it was our stated aim to use locally available materials/resources with a view of value addition, to benefit local areas in the long term. The consortium members have shared facilities (equipment in particular) for analysis and other educational materials among the consortium and more widely, under the signed agreement. This has created an easy route to facility access, especially given that equipment is very expensive and not all universities can afford to acquire the necessary modern equipment or maintain it. Consortium members also had the rights to visit any of the consortium member facilities and make use of any resources, such as libraries, equipment and so forth, where they also had the opportunity to use and be trained on new facilities.

The consortium aimed to maximize value for money throughout the entire project with strict equipment purchasing rules of multiple quotes before purchasing through the best supplier. This was further achieved by attempting to purchase in the UK or a major African country such as South Africa, which through their market size are often more competitive as they host local suppliers. Furthermore, the consortium decided to prioritize the most essential resources for the projects and small equipment, to make sure all basic requirements for the project were catered for. Students were encouraged to make use of existing analytical tools/equipment within the consortium member institutions to carry out their analysis and data acquisition, to make sure that the research deliverables were met even within a limited budget that could not cater for the purchase of major equipment. For example, the consortium decided to buy laptops for the students only, and not for the supervisors or wider group, in order to save money for other much-needed items for the project that would benefit the wider group and not duplicate between institutions.

The consortium’s management committee consisted of the UK and African principal investigators (PIs), one other academic colleague from each institution and the project manager (PM), chaired by the UK PI. Two plenary meetings of the management committee were held each year, with other decisions made by email correspondence and Skype/Zoom/Teams. Most of the key decisions were approved by the whole consortium by consensus, following our initial PI meeting in the spring of 2015, although executive decisions were sometimes made on more minor issues. The UK PI, the chair of our meetings had the deciding vote should the other members be equally divided, although, in practice, it has not been necessary to use a formal vote. The experimental and computational research projects that were carried out primarily in the African participant universities (but with access to the world-class UK facilities as and when required) were designed and harmonized in the conception stage, but refined after the grant had been awarded and the students recruited. All students have spent research visits in partner laboratories, both via South–South and North–South exchanges, and were co-supervised by both African and UK academics, even though individual institutional PIs were responsible for their local projects. If by equitable partnership we mean fair and equal participation in the research agenda and decision-making process, then we consider that our consortium has achieved this objective.

The research specifically undertaken by each partner is summarized below.

### 3.1. Kwame Nkrumah University of Science and Technology

Experimentally guided density functional theory (DFT) calculations have been carried out to elucidate the reaction mechanisms of the depolymerization of lignin from biomass using lignin models and base catalysts, Fe/Ru-xantphos catalysts and Ir/Co-pincer catalysts. This work has opened up the development of novel catalysts for the production of chemical feedstock/platform chemicals or led to sustainable fossil fuel replacements.

In a second research strand into the alkali metal-dosedferrierite group of zeolite minerals (FER structures) to maximize its catalytic potential for the conversion of methanol to hydrocarbons (HCs), DFT and MD calculations were used to examine methanol–methanol and zeolite–methanol interactions with varying methanol loadings and to clarify the nature of the agglomeration of methanol complexes in FER channels based on the FER internal topologies. The reaction mechanism for the methanol to HC conversion over zeolite H-FER together with alkali metals as counter-ions, i.e. Li, Na and K, was also investigated to explore the nature of the reactivity and selectivity of the catalysts at the Lewis acid sites or transition metal centres.

No significant changes needed to be made from the original aims and objectives. Two PhD degrees were awarded, one student is currently a postdoctoral researcher in South Africa and the other is working in a government policy-making position in Nigeria.

### 3.2. University of Botswana and Botswana International University of Science and Technology

The research of two PhD students has focused on the design and execution of experimental hydrogenation reaction procedures to improve the quality of biodiesels from different non-edible plant feedstocks. The research findings showed that the nature of the catalysts used in the hydrogenation reactions significantly affects not only the rate of the reactions, but also the selectivity of the functional groups reduced in the hydrogenation process. We were thus able to select the type of catalyst needed, the length of the hydrogenation process and the reaction conditions to be used to obtain our desired products.

At a colleague university in Botswana, BIUST, an associated part-time student and university lecturer, Nyepetsi supervised by Mbaiwa, has focused on ‘Biodiesel production from beef tallow using non-conventional catalysts’. Tallow, here obtained as a waste product from the local cattle industry, provides two main challenges in biodiesel production, the first being its slower rate of reaction compared to vegetable oils, which was addressed through the use of co-solvents in the process. Second, biodiesel produced from tallow has poor flow properties and which we sought to improve through the use of metal catalysts.

Three PhD degrees were awarded. Two students are now lecturers at their home institutions in Botswana and one students is a postdoctoral researcher at North-West University in South Africa.

### 3.3. University of Namibia

One PhD student has focused on the investigation of zinc-based materials to improve their capacity to photocatalytically split water for clean hydrogen production. The outcomes of this research can now be used to assist and guide on new applications of zinc-based materials, where these products can no longer be used in their raw forms, but rather as processed products.

In another PhD project, copper oxide materials were investigated for similar photocatalytic activities. This work is still in progress, but one scientific article has already been published from the research.

So far one PhD degree has been awarded, the graduate now being a lecturer at her home institution. The other student has taken two periods of maternity leave during her PhD studies and is currently completing her doctoral thesis for submission later in the year. Both these PhD students were already working at the University of Namibia and were given study leave to upskill and obtain PhD degrees, with will benefit not only themselves, but also the future generations of university students that they will be teaching.

### 3.4. Cardiff University

At CU, a PhD student, Živković, funded by CU to be associated with the consortium, has focused on the computer modelling of the structures and properties of a number of copper and ZnO materials, to complement the experimental work in UNAM. In addition, he spent an extended period in UNAM to deliver a computer modelling course to a class of PhD and MSc students to enable them to use computer equipment donated to UNAM, UB and KNUST via the consortium by the CHPC in South Africa.

One PhD degree was awarded; the researcher continued his career with a postdoctoral position in The Netherlands, from where he remained active within the consortium. He has recently been awarded an independent research fellowship in Germany.

## 4. Challenges during the programme

Chem4Energy has been faced with a number of challenges in the delivery of the research collaboration and training, particularly in the earlier years:

— In the early stages, the procurement process used to be a bottleneck. However, having spent time working with the finance departments of CU and the partner institutions, we managed to solve these issues. Fortunately, by the time of the outbreak of COVID-19, most of the procured materials had already been received or at least ordered. Another issue related to equipment procurement was the long delivery times and, even more so, the lengthy bureaucratic delays suffered around import formalities, where equipment could literally spend months in depots before being released to the universities.
— Not surprisingly, training activities and joint research between CU and the African partner institutions have been hampered by the COVID-19 pandemic. This has been especially true for planned researcher exchanges. However, with the speedy adoption of online exchanges, these negative effects have been limited as much as possible. In turn, such techniques have enabled this consortium to ‘meet’ more often than we used to do, thereby providing strong support to the students, and making sure that no researcher or institution was disadvantaged more than was absolutely unavoidable by the pandemic. The computational research in particular was much less affected, by providing remote access to the students to CHPC and CU high-performance computing facilities and library resources as CU visiting students. In addition, some of the experimental students took the opportunity to diverge into carrying out some complementary computational work, which ensured that they could continue their research and added to their skills.
— Just before the UK lockdown, we had to urgently repatriate three students back to their home institutions (2 × UK to Ghana; 1 × Ghana to Botswana) to complete their work remotely. The UNAM students have had to cancel their summer research visit to Swansea University, where they were going to carry out crucial experimental work and testing. Instead, this work has been re-designed and completed at BIUST and the Botswana Institute for Technology Research and Innovation (BITRI) who have made available their facilities to the students. Neither of the UB students has been able to continue to visit the University of Johannesburg (UJ) for the research activities they needed to carry out there as relevant facilities are not available in UB, but they have since caught up and have both graduated with their PhDs.
— We had to cancel the full consortium meeting in Accra, Ghana, in April 2020, where we had planned training sessions with a career adviser from North-West University (NWU), South Africa, plus CV writing exercises and mock interviews. However, these activities were completed at the final full consortium meeting held in South Africa in April 2022. Unfortunately, our planned final meeting at UNAM, where we had planned to hold a workshop with industrial and other external partners to discuss the continuation and sustainability of the research programme and consortium, has also had to be cancelled, which is likely to affect the long-term sustainability of the consortium and collaborative research, as we have not had the opportunity to meet and build links with potential industrial supporters.
— Another challenge experienced by one of our PhD students at UNAM was already alluded to above, i.e. that Sheehama could not complete within the funded period due to two periods of maternity leave. However, she has continued with her PhD research locally and will still be able to benefit from the wider consortium advice and access to resources at partner institutions, in particular BIUST in the same region.
— To a lesser extent, the sourcing and delivery of required equipment to the African partners remained difficult, expensive and time-consuming, although, in the end, all partners received the equipment they needed for the students to execute their research and to ensure sustainability of the African laboratories.

Lessons learnt from the pandemic concerning the implementation of contingency planning are vital to the success of future partnerships. Skills and knowledge transfer between partners in future will require not only the funds for researcher exchanges but also flexibility in increasing fair access to online tools between all partners, with funds specifically set aside for such endeavours. The consortium converted a significant portion of the travel budget to student bursaries to allow the African partners to complete the research objectives of the project without significant travel abroad and use the facilities either in-house, at BITRI or UJ in South Africa to analyse the final samples and obtain relevant data.

The passing of Prof. Richard Tia from KNUST in November 2022 from liver cancer had a major impact on the students and the community. Tia was an associate professor of chemistry and a computational and theoretical chemist in the College of Science’s Department of Chemistry and had been a very active partner even before the Chem4Energy consortium had been formed. His loss is still felt and we would like to dedicate this publication to his memory.

## 5. Outputs and successes from the programme

The Chem4Energy research has already led to 15 publications by the seven PhD students graduated from the programme, all of whom stayed in academia or science-based employment, and over 50 publications by the wider scientific community associated with the consortium. Highlights of the programme include:

— Active collaborations by students with experienced academics have expanded the networking possibilities between the partners and created additional collaborations with other researchers and institutions in Africa, the UK and even further afield (The Netherlands, USA).
— Students have been exposed through short training and extended research visits to partner universities.
— Capacity building in the field of renewable energy is gaining momentum (including encouraging the inclusion of women).
— Constructive regular seminars and sandwich supervision have contributed immensely to student progress.
— The development of the students has led to their increasing confidence in undertaking high quality, often quite independent research and presenting their research outcomes to a scientific community.

The KNUST computational group has been a major beneficiary of the RS Africa capacity-building research collaboration initiatives, which through RS/Leverhulme and RS/DFID funding has added materials modelling capability to the existing molecular modelling expertise, resulting in the training and graduation of five PhD and two MSc students and the provision of an institutional HPC facility for the computational chemistry group and other KNUST researchers. In addition, participation in the consortium has led to continuing access to the internationally competitive resources at CHPC South Africa, facilitated through the existing collaborative contacts of the UK PI in South Africa. KNUST students have also had the opportunity to spend an extended research visit to the CHPC facility, where they have been given training on hardware and software.

Fostering south–south collaborations for the sustainability of the consortium was one of the major goals of the Chem4Energy programme and although there was no funding for South African partners in our original consortium, a separate grant under the UKRI Newton fund obtained by the UK PI with South African partners, fostered strong links between the ACBI consortium and the Newton partners, forming a strong collaboration between UK, Botswana, Namibia, Ghana and South Africa institutions. Specifically, the hosting of two conferences at NWU for both programmes established mutually beneficial links between the Chem4Energy African partners and research groups in three strong universities in South Africa, i.e. the University of Limpopo, the University of Cape Town and NWU. Within this extended partnership, computational training was provided to all students by the CHPC in Cape Town, whose facilities continue to be made available to all the partners involved. Experimental research was conducted at UJ, where again the research collaborations and equipment sharing are ongoing. These examples of strong south–south links, built up during the project, have continued after the consortium’s RS/DFID funding finished.

A number of high-impact publications has also increased the scientific standing of CU. We have edited a thematic issue of the *South African Journal of Chemistry* (Africa-UK Partnership for the Computer-aided Development of Sustainable Catalysts, editors: D. Santos-Carballal, C. G. C. E. van Sittert, N. H. de Leeuw (2021)) [7], with publications from all groups, together with a number of South African PhD exchange students, which is an excellent collection of the research carried out over the duration of the project.

During the ACBI award, one of the goals was to train the students to be ready for employment upon the completion of their PhD studies. To this end, general and technical training has included:

— Virtual consortium meetings to train students to present at an international conference and to use online facilities.
— Face-to-face participation at the Cardiff annual meeting, RSC Faraday Discussion meeting in Cape Town, and FASC conference in Gaborone, to experience presenting in person at an international conference.
— CV writing and mock interviews to prepare the students for employment applications.
— Training in the use of high-performance computing facilities at both CU and their home institutions using the CHPC facilities in South Africa.
— Other skills focused on during training activities included critical thinking, problem solving, adaptability, teamwork, attention to detail, time and data management.

## 6. Published research

In this section, we provide a brief summary of the research outcomes from the PhD work of the students supported on the ACBI grant in the Chem4Energy consortium.

### 6.1. Kwame Nkrumah University of Science and Technology

#### 6.1.1. Understanding methanol diffusion and framework methylation mechanism in Brønsted acid and alkaline metal-modified zeolite topologies: a DFT and MD study

Despite climate change and the global energy crisis, renewable energy still accounts for less than 27% of the total energy consumption of the world [8]. In this work, we have investigated the use of aluminium silicate crystals in the conversion of alcohols to short-chain HCs to be used as fuels but also as useful precursor molecules for the chemical industry [9]. The lack of agreement as to optimal commercially relevant parameters, such as temperature, Brønsted acid site distribution and the influence of alkali metals on conversion rates still leaves open questions when considering the methanol-to-hydrocarbon (MTH) process [10].

Our study sought to determine the effect of the topology of zeolites formed from cheap kaolin sources (Ghana) on the diffusion of feedstock compounds like methanol as it is converted. The study also investigated the most likely adsorption conformation in the selected zeolites to inform the nature of the HC-pool mechanism [11] and subsequent control of the predetermined desired products. MD simulations, DFT calculations and quasi-elastic neutron scattering (QENS) analysis were used in the understanding of the radial distribution function (RDF) and hydrogen bonding to elucidate the nature of the interaction between alcohol molecules and the zeolite catalysts. The MD simulations were used to model the zeolites Beta, MFI and FER with experimentally determined acid sites in varying sections of the selected zeolite topologies. The studies were conducted using the DL_POLY package [6] and the visualizations of the production runs and trajectories were obtained with visual MD [12]. The optimized structures and energetics of zeolite FER were calculated using DFT as implemented in the Quantum Espresso package [13]. Further calculations were carried out using only the reaction pore of zeolite FER with the Gaussian 09 package [1] using the M06 functionals and 6-311G basis set [14]. The transition state structures were verified to have only one imaginary frequency.

**Figure 1.**
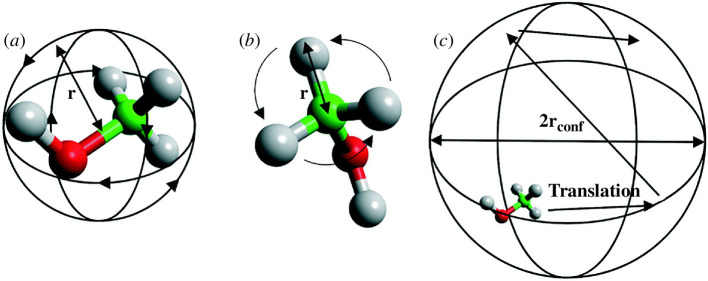
A methanol molecule is shown in the diagram (*a*) spinning isotropically, (*b*) rotating uniaxially or in a jump manner and (*c*) diffusing within a sphere. (Reproduced from [15] with permission from the Royal Society of Chemistry.)

**Figure 2.**
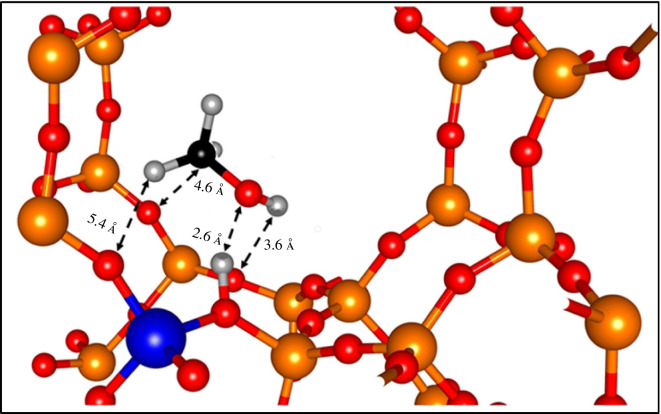
Image of the side-on configuration of methanol adsorption in zeolites Beta and MFI, where the molecules are aligned according to the RDF of the atomic pairs *Om-Ha, *H-O, *Hm-O and *C-O. Element colour code: O (red), Si (orange), Al (blue), H (grey), C (black). (Reproduced from [16] with permission from MDPI.)

The MD simulations yielded diffusion coefficients within the range of 2.0–10.0 × 10^–10^ m^2^ s^–1^ for zeolite MFI, indicating significant mobility of the methanol. High temperatures considerably reduced the contact time between methanol and the acid sites in zeolite Beta compared to zeolite MFI, with more methanol clustering in zeolite Beta than in zeolite MFI. Confined dynamics which comprise isotropic rotation, and uniaxial twists were also observed in zeolite FER, as shown in figure 1. Again, when considering RDF, transient movement of the methanol molecule was determined, when adsorbed at the Brønsted acid sites of the zeolite catalysts, shown in figure 2. The methanol molecule preferred the side-on orientation rather than the end-on orientation, especially in zeolite MFI, which indicates a high probability of cleaving the C–O_m_ bond.

The capacity of the counter-ion, required for charge balancing, to coordinate methanol in the reaction pore determines the thermochemical stability of adsorbed methanol in alkali metal-substituted FER, according to DFT studies [17]. Thus, when alkali metals are used as counter-ions, methanol was observed to agglomerate differently depending on the metal; the methanol clusters are created by direct interactions between the metal and the methanol hydroxyl oxygen O_m_. At higher loadings of four methanol molecules per unit cell (mpuc), the Li counter-ion interacts directly with up to two hydroxyl oxygens of methanol, whereas the Na counter-ions contact directly with three methanol molecules, again through their hydroxyl oxygens at 4 mpuc. The capacity of metals to receive electron pairs determines how many methanol molecules react directly with the metal counter-ions in this way. This behaviour was in close agreement with data from QENS analysis of methanol diffusion in zeolite FER samples that were synthesized from kaolin sources and contained alkali metal counter-ions [18].

Using DFT calculations, the impact of intermediates after dehydration of the zeolites on the methanol adsorption energies was examined. Since the presence of water molecules facilitates the adsorption of methanol, polarity differences between dimethyl ether and water were found to be critical to the stability of adsorbed methanol. Only at low loadings was substantial methanol adsorption with water noticeable. Apart from Na-FER, the addition of dimethyl ether often reduced the stability of adsorbed methanol without affecting the C–O_m_ stretch for the methanol complexes, regardless of mixing ratios. The findings implied that methanol should be added at high temperatures to enhance diffusivity and at low concentrations to minimize methanol–methanol interactions to optimize the catalytic potential of alkali metal-dosed FER.

**Figure 3.**
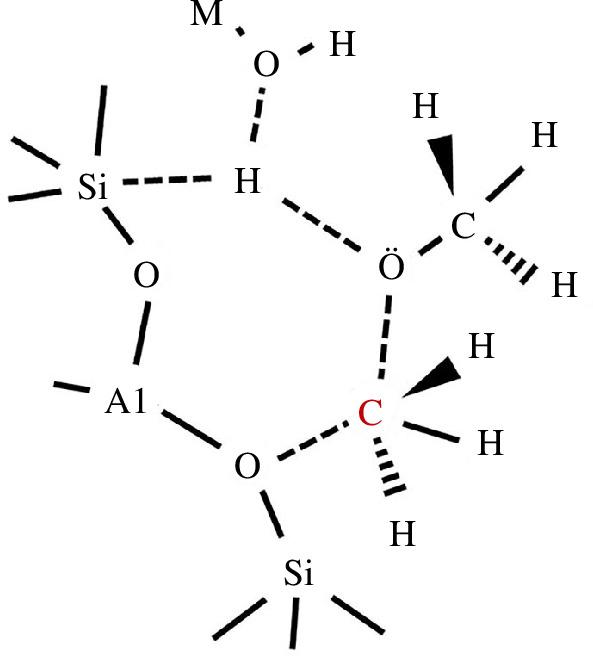
Transition state that leads to the formation of dimethyl ether after methanol dehydration.

Subsequently, the methylation reaction process in zeolite FER with alkali metal counter-ions was investigated. The findings showed that when the atomic radius of the alkali metal cations increased, the adsorption stability gradually reduced. The methanol adsorbate undergoes C–O_m_ bond cleavage after being absorbed at the acid site, resulting in the production of a metal hydroxide and a surface methoxy group. The metal hydroxide extracts the hydroxyl hydrogen from the injected methanol molecule when it is introduced into the pore. As seen in figure 3, the free methyl groups combine with the surface methoxy group to form dimethyl ether and a water–metal complex with an energy of −42.1 kcal mol^−1^.

In conclusion, the zeolite architecture, which includes the interconnected sinusoidal and straight channels of zeolite MFI, plays a crucial role in the optimal balance of methanol adsorption and mobility during the MTH process. Particularly at temperatures lower than 473 K, the MD simulations confirmed that the suggested end-on and side-on topologies are components of the same MTH reaction process. Additionally, we found that particularly at low methanol loadings, methanol dehydration is simpler in more constrained zeolite pores (10-membered rings). Hydrogen continues to be the most preferred counter-ion for methanol dehydration and the MTH process overall, even though methanol clustering is shown with all of the alkali metals that were examined.

#### 6.1.2. Mechanistic studies on the homogeneous base- and transition metal-catalysed cleavage of aryl–ether linkages in lignin: a DFT study

The β-O-4 linkage is pervasive in lignin and selective cleavage of this linkage using environmentally friendly and cost-efficient methods is an important step in the sustainable depolymerization of lignin towards the conversion of lignocellulosic biomass to platform chemicals and fuels. DFT calculations at the M06 level have been used to elucidate the mechanism of the catalysed cleavage of a β-O-4 linkage using a series of base catalysts (NaOH, KOH, LiOH and Na*^t^*Obu), a xantphos-containing ruthenium and iron catalyst, as well as a pincer-containing iridium and cobalt catalyst. Two types of the β-O-4 linkage were studied: a C2 type, which contains no γ-carbinol group, and a C3 type, which contains a γ-carbinol and is more representative of the actual linkage in lignin. By changing metals and ligands and assessing the electronic structures of the reaction system, the factors that contribute to the chemical reactivity and selectivity were explored. The molecular-level insights obtained from this work can help in the design of sustainable atom-economic catalysts, which are active towards the valorization of lignin for the production of value-added fine chemicals and fuels.

**Scheme 1.**
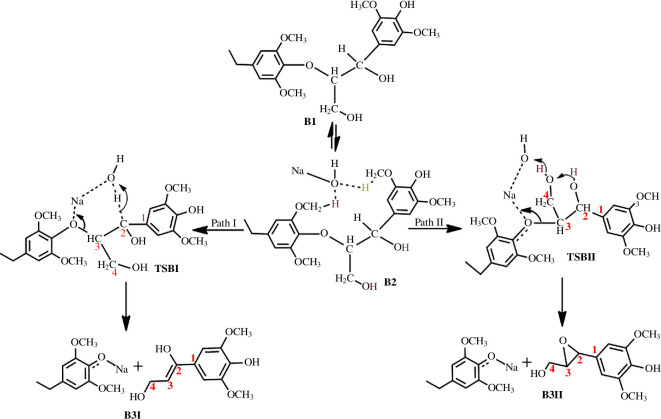
Mechanism for the base-catalysed cleavage of the β-O-4 linkage found in lignin proposed by Mensah *et al*. [20] with permission from *Frontiers in Chemistry*.

DFT (M06) methods in conjunction with the 6-31G* basis set showed that the cleavage of the ether bond in the C2 substrate using LiOH^−^, NaOH^−^, KOH^−^ and NaO*^t^*Bu proceeds via a 6-membered transition state in which the hydroxide ion deprotonates the α-carbon, thereby promoting the C–O bond cleavage, which is consistent with the mechanism proposed by Roberts *et al.* [19]. The ether bond cleavage involving KOH has the lowest activation barrier of 6.1 kcal mol^−1^ with a calculated rate constant of 2.1 × 10^8^ s^−1^. Cleavage of the C3 substrate is found to proceed via two pathways: an enol-formation pathway and an epoxide-formation pathway (Scheme 1 and table 1). The first path is the thermodynamically favoured pathway, which is similar to the pathway for the C2 substrate, and is the preferred pathway for the isolation of an enol-containing monomer. The second path is the kinetically favoured pathway, which proceeds via an eight-membered transition state involving a hydrogen hopping event (as shown by TSBII in Scheme 1), and is the preferred pathway for the isolation of an epoxide-containing monomer, as observed by Mcdonough [21] and akin to the Grotthus mechanism proposed by Agmon [22]. The KOH-catalysed reaction also has the lowest activation barrier of 10.1 kcal mol^−1^ along the first path and 3.9 kcal mol^−1^ along the second path, with calculated rate constants of 2.4 × 10^5^ s^−1^ and 8.6 × 10^9^ s^−1^, respectively.

**Table 1.** Relative energies for the catalysed cleavage of the C3 β-O-4 substrate using KOH, LiOH and NaO*^t^*Bu. All energies are measured in kcal mol^−1^. (Reproduced from [20] with permission from *Frontiers in Chemistry.*)

base	*E*_a_ (TSBI)	*E*_a_ (TSBII)	*E*_f_ (B3I)	*E*_f_ (B3II)
KOH	10.1	3.9	−33.0	−21.1
LiOH	20.7	11.4	−27.6	−15.7
NaO*^t^*Bu	16.4	11.3	−27.8	−15.9

**Scheme 2.**
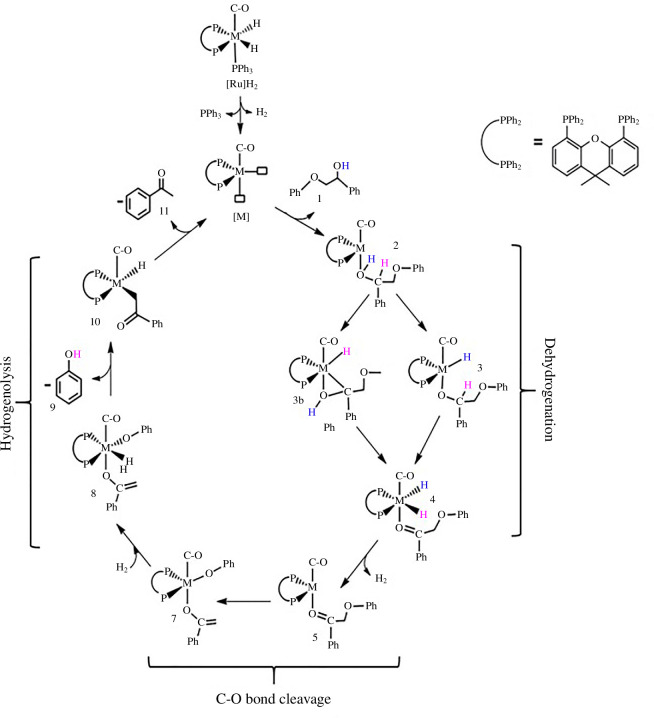
Mechanism for the Fe- and Ru-xantphos catalysed cleavage of the β-O-4 linkage found in lignin proposed by Mensah *et al*. [20] with permission from *Frontiers in Chemistry*.

Using the M06/6-31G(d)/Lanl2dz method, we determined the kinetically favoured pathway for the dehydrogenation step. With Ru, this pathway begins with an oxidative addition reaction followed by β-hydride elimination leading to the formation of a dihydride complex, as observed by Chmely *et al*. [23]. However, in the Fe system, the dehydrogenation step begins with a β-hydride elimination reaction followed by an oxidative addition to the metal centre which leads to the formation of a dihydrogen complex (Scheme 2).

**Figure 4.**
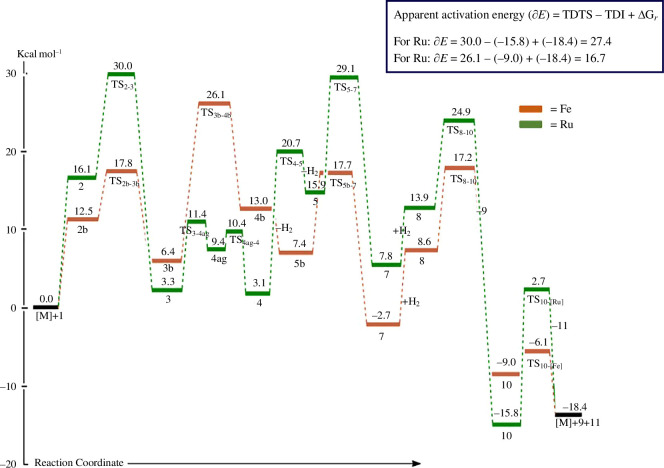
Energy profile of the dehydrogenation of 1 using [Fe] and [Ru].

Tandem dehydrogenation/hydrogenolysis of the C2-β-O-4 model compound is kinetically controlled, having an apparent activation energy of *δE* = 24.7 kcal mol^−1^ and a rate constant of 5.1 × 10^−8^ s^−1^ with [Ru], while with [Fe] they are 16.7 kcal mol^−1^ and 3.5 s^−1^, respectively. Cleavage of the C3-β-O-4 model compound is also kinetically controlled, *δE* of 34.7 and 14.5 kcal mol^−1^ for [Ru] and [Fe], respectively, and calculated rate constants of 2.3 × 10^−13^ s^−1^ and 145.7 s^−1^ for [Ru] and [Fe], respectively. Thus, the [Fe] system is more active towards the catalytic cleavage of the C2 model compound, being kinetically favoured by 10.7 kcal mol^−1^ over [Ru], and even more active towards the cleavage of the C3 substrate, being kinetically favoured by 20.2 kcal mol^−1^ [24] (figure 4).

A M06/6−311G**/LANL2TZ study of the cleavage of lignin 2-phenoxy-1-phenylethanol by using iridium and cobalt pincer (^ipr^PCP)-Ir, (^ipr^PCOP)-Ir, (^ipr^PCP)-Co and (^ipr^PCOP)-Co complexes (where PCP = κ3-C6H3-2,6-[CH2P(t-Bu)2]2) showed that both iridium and cobalt are found to be active towards the cleavage of the β-O-4 linkage with rate constants of 44.7 s^−1^ and 5.1 × 10^6^ s^−1^, respectively [25]. The observed selectivity for the initial C–H addition reaction pathway as observed in experiments when using the (^ipr^PCP)Ir catalyst for the cleavage of simple ether bonds [26] was confirmed with a kinetic preference of 16.8 kcal mol^−1^ over the ‘direct C–O insertion’ pathway. However, the cobalt catalyst showed a preference for the direct C–O bond addition pathway and a mechanism, which involves an initial dehydrogenation step, consistent with observation by Nichols *et al*. [27], with a kinetic preference of 15.7 kcal mol^–1^ over the ‘initial C–H addition’ pathway. A two-state reactivity occurs along the preferred pathway for the cobalt-catalysed reaction.

These detailed insights into the thermodynamics and kinetics of the cleavage of aryl–ether linkages has shown that KOH, NaOH, Ru-xantphos, Fe-xantphos, PCP-type iridium and cobalt complexes are all active catalysts for the selective depolymerization of the β-O-4 linkage in lignin.

### 6.2. University of Botswana

#### 6.2.1. The experimental exploration of pyrazolyl complexes as catalysts in the generation of sustainable fuels

**Figure 5.**
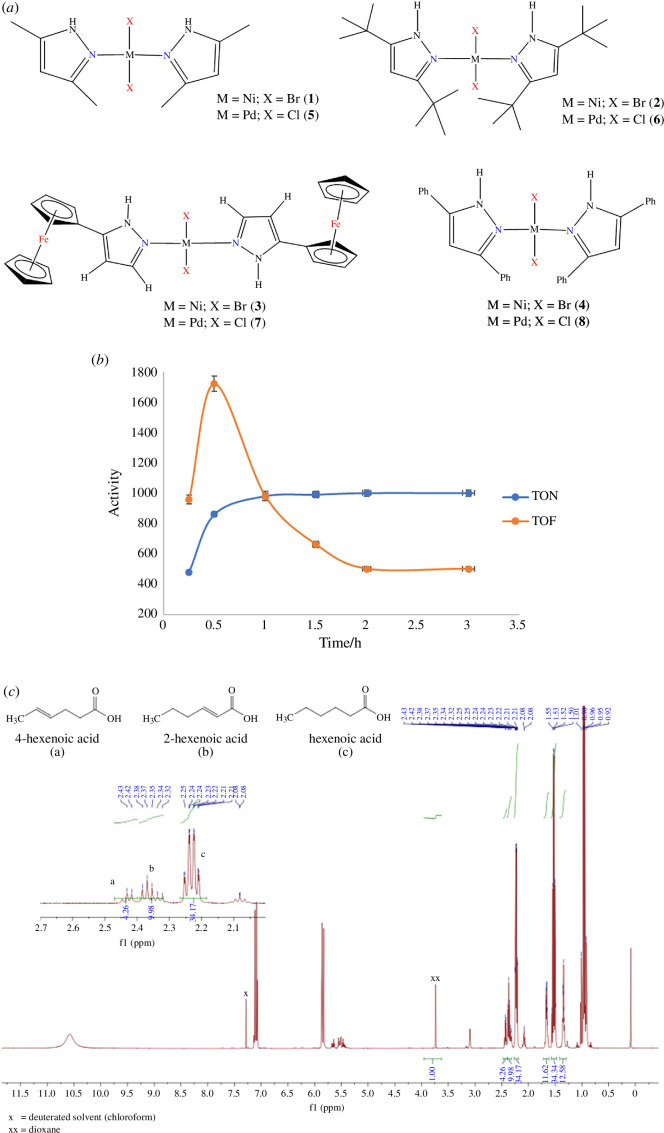
(*a*) The synthesized pyrazolyl-based Ni(II) and Pd(II) complexes for the hydrogenation reaction. (*b*) Catalytic activities of the pyrazolyl complexes for the hydrogenation reactions. (*c*) Representative ^1^H NMR spectrum of the hydrogenation of sorbic acid with molecular hydrogen to produce the intermediates.

Concerns about climate change and high demands for energy have sparked a keen interest in the development of other sources of fuels such as biodiesel. Given its well-known benefits, e.g. non-toxicity, sourcing from renewable feedstocks and biodegradability, biodiesel is a promising substitute for conventional petroleum-based diesel fuel [28–31]. One method to enhance this renewable fuel is to hydrogenate free-fatty acid methyl esters (FAMEs) [31]. Our work has focused on the synthesis and characterization of pyrazolyl-based Ni(II) and Pd(II) complexes to hydrogenate simple molecules and biodiesels (figure 5). The catalytic activities of pyrazolyl nickel complexes were investigated for the hydrogenation of 1-octene to *n*-octane. The increase in the reaction time, pressure and temperature greatly affected the hydrogenation of 1-octene with a pyrazolyl nickel(II) complex, having the highest turnover frequency (TOF) of 1720, 1960 and 2000 h^–1^, respectively. The recyclability of the pyrazolyl complex as a catalyst for the hydrogenation of 1-octene was performed on the subsequent addition of the substrate (1-octene) after each run for 1 h. With up to five efficient recycling cycles without causing appreciable losses in the process, the catalyst demonstrated exceptional recyclability. The influence of metal centres on the hydrogenation of sorbic acid, using formic acid as a source of hydrogen and molecular hydrogen was also investigated, where the pyrazolyl palladium(II) complexes were found to be more efficient than their corresponding nickel(II) counterparts. In addition, all the complexes studied as pre-catalysts for the catalytic hydrogenation of sorbic acid demonstrated considerable conversions (i.e. far more than 50%) and efficiency [32].

Finally, the selective and/or partial hydrogenation of free-FAME was used to assess the catalytic activities of the pyrazolyl-based Ni(II) and Pd(II) complexes. Methyl linoleate (ML) can be selectively and partially hydrogenated by the synthesized mononuclear complexes, which show notable catalytic activities [31]. The hydrogenation of a typical biodiesel by the pyrazolyl Pd-complex was followed by ^1^H NMR spectroscopy [31]. The pyrazolyl-based complexes were also tested to hydrogenate biodiesel (FAME) produced from native and non-edible Botswana plant seed oils. The project’s significant accomplishment is in the production of liquid and solid fuels from these oils [31].

#### 6.2.2. The transfer hydrogenation of cinnamaldehyde using homogeneous cobalt(II) and nickel(II) (*E*)-1-(pyridin-2-yl)-*N*-(3-(triethoxysilyl)propyl)methanimine and the complexes anchored on Fe_3_O_4_ support as pre-catalysts: an experimental and *in silico* approach

**Figure 6.**
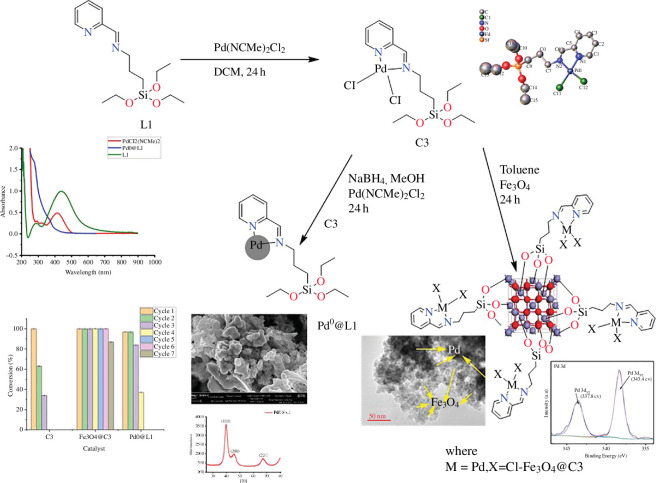
General scheme for the synthesis and characterization of the heterogeneous catalysts Fe_3_O_4_@C3 and Pd^0^@L1 from the homogeneous catalyst C3.

Metallic nanoparticles have sparked significant interest owing to their size-dependent properties and zero-valent oxidation states/electronic configuration, which leads to excellent properties for applications in catalysis [33]. Nanocatalysts comprise a wide range of compounds where a ligand is incorporated into the nanoparticles to influence the activity and selectivity. Ligands can stabilize nanoparticles as well as reduce aggregation [34]. For example, the lone pairs in nitrogen atoms can attach to the surface of palladium nanoparticles, yielding stabilized nanocatalysts with attractive catalytic properties [35,36]. In the literature, palladium nanoparticles stabilized with phenanthroline ligands on polyethylene have been reported as active hydrogenation catalysts, giving turnover numbers (TON) in the range of 422–448, conversions of 100% and 100% selectivity towards the hydrogenation of C = C bonds in different alkene substrates (reaction conditions: 10 mmol substrate, 5 mg catalyst, stir, H_2_ balloon) [37]. In addition, nanosupports such as metal oxides can be used to incorporate homogeneous catalysts, which yields highly recyclable catalysts suitable for a biorefinery in a green catalysis process [38]. This work aims to demonstrate the synthesis and characterization of Pd(0) nanocatalysts stabilized with bidentate nitrogen binding ligands as novel catalysts for the hydrogenation of furfural to produce furfural alcohol, which is an intermediate on the route to several fine chemicals (figure 6). These catalysts will be compared with other known catalysts to establish their catalytic efficiency. From scanning electron microscopy (SEM) and transmission electron microscopy data, the catalysts were confirmed to be spherical nanoparticles with an average size of 5 nm, and the functional groups, such as C = N and Si–O, of the ligand L1 were also observed from the IR spectra. The EDX spectra show all the expected elements in the two catalysts, while from XPS, the binding energies corresponding to Pd in the Fe_3_O_4_@C3 were observed as Pd3/2 and Pd5/2 doublets at 337.8 and 343.4 eV, respectively, which confirms the successful synthesis of the desired catalysts. The two catalysts (Pd^0^@L1 and Fe_3_O_4_@C3) convert furfural to furfural alcohol only, without hydrogenating the internal olefinic bonds in the furfural molecule, giving the highest TOF of 111 h^–1^. The catalysts are recyclable over four catalytic cycles, with catalyst deactivation only detected in the fifth and sixth cycles.

#### 6.2.3. Liquid-phase decarboxylation of fatty acid methyl esters: towards improving the flow properties of beef tallow-based biodiesel

**Figure 7.**
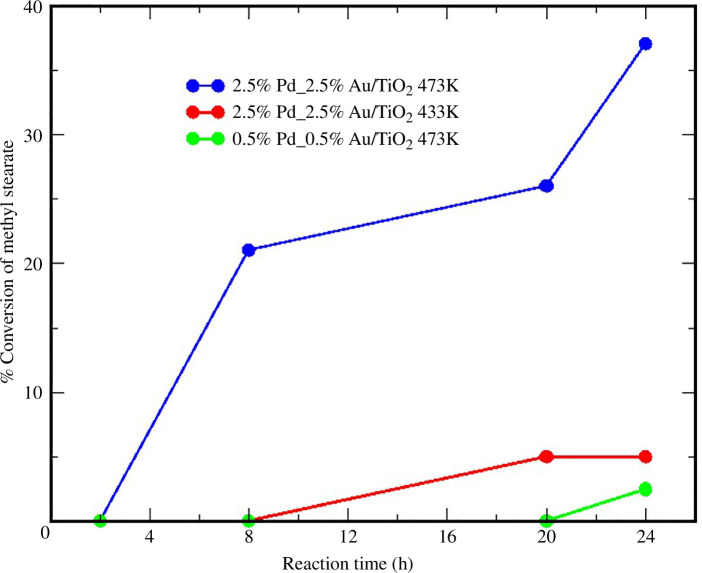
Conversion of methyl stearate as a function of time and temperature.

In this work, we have investigated whether tallow could be a viable source of biodiesel. Botswana possesses a large cattle population, and the beef industry produces tallow as its byproduct. This tallow can be used as a feedstock for biodiesel production through the transesterification process. However, one of the primary issues with beef tallow biodiesel is poor flow properties caused by crystallization at relatively high temperatures [39,40]. Other constraints include high viscosity, pour point, cloud point and acidity values, caused by the relatively high composition of saturated FAMEs like methyl stearate and palmitic methyl ester. The high oxygen content in biodiesel is also responsible for these poor flow properties. One of the ways of improving the poor flow properties is by converting FAMEs to HCs, which can be achieved by the decarboxylation of FAMEs. Our research is aimed at improving the flow properties of beef tallow biodiesel by liquid-phase hydrodeoxygenation (HDO) of methyl esters [41,42]. This method was selected because the products obtained are HCs and can be used with current automotive engines without modification. The catalysts 0.5%Au−0.5%Pd/TiO_2_ and 2.5%Au−2.5% Pd/TiO_2_ were synthesized using a modified wet impregnation method [43], followed by 4 h calcination in 5% H_2_ in argon at 400°C with a heating rate of 10°C min^−1^ before characterization of the prepared catalysts by BET, SEM-EDX and XRD. HDO reactions using the prepared catalysts on methyl stearate were carried out in a batch reactor and with hydrogen at a pressure of 3.5 MPa. The highest methyl stearate conversions were observed when 2.5%Pd−2.5%Au/TiO_2_ was used as a catalyst, with 40% conversion achieved in only 24 h (figure 7). The main products of the HDO process were found to be octadecane and heptadecane with octadecanol and stearic acid produced in smaller quantities.

### 6.3. University of Namibia and Cardiff University

#### 6.3.1. Zinc sulfide and ZnO thin films

Metal sulfides, including zinc sulfide (ZnS), are semiconductor photocatalysts that have been investigated [44] for the photocatalytic degradation of organic pollutants as well as their activity during the hydrogen evolution reaction and water electrolysis. However, devising ZnS photocatalysts with a high overall quantum efficiency has been a challenge owing to the rapid recombination rates of the charge carriers. Various strategies, including the control of size and morphology of ZnS nanoparticles, have been proposed to overcome these drawbacks. In this work, ZnS samples with different morphologies were prepared from zinc and sulfur powders via a facile hydrothermal method by varying the amount of sodium borohydride used as a reducing agent. The structural properties of the ZnS nanoparticles were analysed by X-ray diffraction (XRD), SEM and X-ray photoelectron spectroscopy (XPS) techniques. All-electron hybrid DFT calculations were used to elucidate the effect of sulfur and zinc vacancies occurring in the bulk as well as at the (220) surface on the overall electronic and absorption properties of ZnS. Considerable differences in the defect level positions were observed between the bulk and surface of ZnS while the adsorption of NaBH_4_ was found to be highly favourable but without any significant effect on the band gap of ZnS. The photocatalytic activity of ZnS was evaluated for the degradation of rhodamine B dye under UV irradiation and hydrogen generation from water. The ZnS nanoparticles photocatalytically degraded rhodamine B dye effectively, with the sample containing 0.01 mol NaBH_4_ being the most efficient. The samples also showed activity for hydrogen evolution, but with less H_2_ produced compared to untreated samples of ZnS. These findings suggest that ZnS nanoparticles are effective photocatalysts for the degradation of rhodamine B dyes as well as hydrogen evolution, but rapid recombination of charge carriers remains a factor that needs future optimization.

ZnO is a versatile and inexpensive semiconductor with a wide direct band gap that has applicability in several scientific and technological fields. In this work [45], we report the synthesis of ZnO thin films via two simple and low-cost synthesis routes, i.e. the molecular precursor method (MPM) and the sol–gel method, which were deposited successfully on microscope glass substrates. The films were characterized for their structural and optical properties. XRD characterization showed that the ZnO films were highly *c*-axis (002) oriented, which is of interest for piezo-electric applications. The surface roughness derived from atomic force microscopy (AFM) analysis indicated that films prepared via MPM were relatively rough with an average roughness (Ra) of 2.73 nm compared to those prepared via the sol–gel method (Ra = 1.55 nm). Thin films prepared via MPM were more transparent than those prepared via the sol–gel method. The optical band gap of ZnO thin films obtained via the sol–gel method was 3.25 eV, which falls within the range found by other authors. However, there was a broadening of the optical band gap (3.75 eV) in thin films derived from MPM.

#### 6.3.2. Zinc phosphate materials for photovoltaic applications

Binary II–V semiconductors are optically active materials [46], possess high intrinsic mechanical and chemical durability, and have electronic properties ideal for optoelectronic applications. Among them, zinc diphosphide (ZnP_2_) is a promising earth-abundant absorber material for solar energy conversion. The resurgence of interest in zinc phosphide compounds as efficient solar absorbers has initiated increasing efforts to improve their stability under humid and oxygen-rich conditions [47]. Zinc phosphides (ZnP_2_ and Zn_3_P_2_) are emerging [48] absorber materials for photovoltaic (PV) applications owing to their abundancy and non-toxic nature. We have investigated the structural, mechanical and optoelectronic properties of both the tetragonal (α) and monoclinic (β) phases of ZnP_2_ using standard, Hubbard-corrected and screened hybrid DFT methods. Through the analysis of bond character, band gap nature and absorption spectra, we show that there exist two polymorphs of the β phase (denoted as β1 and β2) with distinct differences in the PV potential. While β1 exhibits the characteristics of metallic compounds, β2 is a semiconductor with predicted thin-film PV absorbing efficiency of almost 10%. The α phase is anticipated to be an indirect gap material with a calculated efficiency limited to only 1%. We have also analysed and gained insights into the electron localization function, projected density of states and projected crystal orbital Hamilton populations for the analogue bonds between the α and β-ZnP_2_.

**Figure 8.**
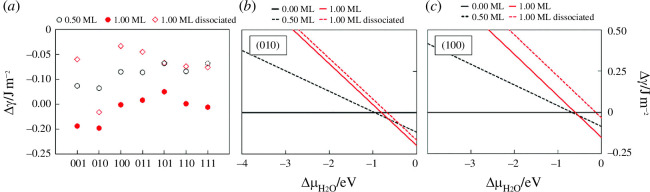
(*a*) Changes in the surface energy for seven low-Miller-index surfaces of β-ZnP_2_ induced by a half and full monolayer (ML) hydration and a full monolayer hydroxylation at 0 K. Because the (111) surface can accommodate three water molecules to form a full ML, the respective data point for lower coverage corresponds to a 0.33 ML and is hence shown in dashed border lines. (*b*) Changes in the surface energy of the (010) surface with respect to the chemical potential of water. (*c*) Changes in the surface energy of the (100) surface with respect to the chemical potential of water. (Reproduced from [48] with permission from the Royal Society of Chemistry.)

We have carried out a comprehensive characterization of the surface structure, composition, stabilities, morphology and electronic properties of both bare and hydrated/hydroxylated low-Miller-index surfaces of β-ZnP_2_ by means of DFT calculations. Mechanistic insights into the fundamental aspects of water adsorption and dissociation, including the adsorption geometries, energetics and structural parameters along the reaction path were characterized systematically. The stabilities of the surfaces under dry and wet conditions were discussed and the predicted phase diagrams for the water adsorption are presented in figure 8.

**Figure 9.**
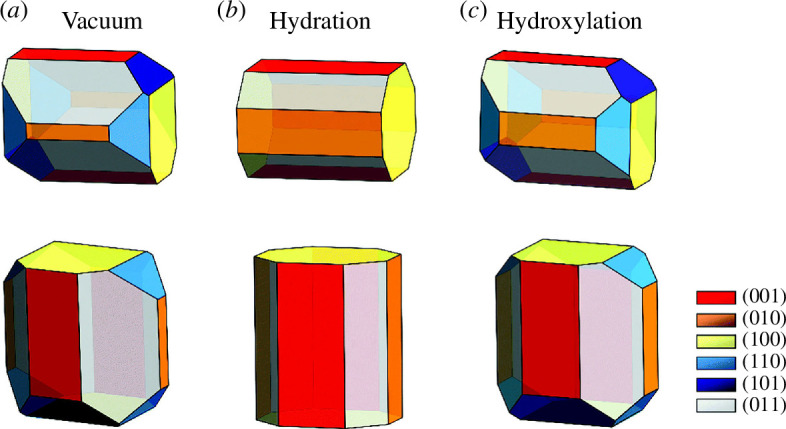
Wulff morphologies of β-ZnP_2_ (*a*) in vacuum, (*b*) after hydration and (*c*) after hydroxylation. (Reproduced from [48] with permission from the Royal Society of Chemistry.)

Using calculated surface energies, we also derived the equilibrium morphology of the β-ZnP_2_ nanocrystals under vacuum and upon hydration or hydroxylation (figure 9). The strength of water adsorption on each surface is found to decrease with the increasing number of water molecules. It is clear from our calculated surface energies that the molecular water adsorption (hydration) affects the stability of the β-ZnP_2_ surfaces more than the dissociative water adsorption (hydroxylation), substantially modifying the equilibrium morphology of β-ZnP_2_ nanocrystals. Atomic-level insights into the origin of the incipient oxidation of β-ZnP_2_ surfaces were provided through analysis of the Bader charges, which revealed that the Zn sites to which H_2_O and OH species are bound undergo oxidation owing to the transfer of charge to the adsorbed species. Adsorption-induced changes to the electronic properties before and after hydration/hydroxylation were characterized by the work function and partial density of states. The results highlight the need for protection of β-ZnP_2_ nanocrystals against possible oxidation in the presence of water through post-synthesis organic functionalization.

Although organic functionalization has been suggested as a promising strategy to passivate zinc phosphide nanoparticles, fundamental atomic-level insights into the adsorption processes and structures at zinc diphosphide (ZnP_2_) surfaces are still lacking. Here, the interactions between 4-aminothiophenol and the low-Miller-index surfaces of monoclinic ZnP_2_ were investigated by means of DFT calculations. A bidentate adsorption mode, in which 4-aminothiophenol binds through both its functional groups via Zn–N and Zn–S bonds, was predicted to be the strongest form of interaction, and monolayer-functionalized ZnP_2_ surfaces were found to be highly stable under adsorbate-rich conditions. Changes in the equilibrium morphology of ZnP_2_ nanocrystallites upon functionalization and effects of humidity were also considered. The insight from our calculations is expected to contribute toward the rational design of ZnP_2_-based materials for PV devices.

#### 6.3.3. Computer modelling of copper oxides

Hybrid DFT has been used to study [49] the phase stability and formation of native point defects in Cu_4_O_3_. This intermediate copper oxide compound, also known as paramelaconite, was observed to be difficult to synthesize owing to stabilization issues between mixed-valence Cu^1+^ and Cu^2+^ ions. The stability range of Cu_4_O_3_ was investigated and shown to be realized in an extremely narrow region of phase space, with Cu_2_O and CuO forming readily as competing impurity phases. The origin of p-type conductivity is confirmed to arise from specific intrinsic copper vacancies occurring on the 1+ site. Away from the outlined stability region, the dominant charge carriers become oxygen interstitials, impairing the conductivity by creating deep acceptor states in the electronic band gap region and driving the formation of alternative phases. This work demonstrated the inadequacy of native defects as a source of n-type conductivity and it complements existing experimental findings.

## 7. Conclusions and future outlook

Our collaborative research programme aimed to train a cadre of African scientists in the use of state-of-the-art computer modelling and advanced experimental techniques applied in materials science and catalysis. The collaborative research was co-created by the teams from all four partner institutions and was chosen to focus on sustainable energy generation using locally available, non-edible and waste materials. In addition to technical training, the comprehensive training programme delivered in the African institutions included generic and power skills training.

Altogether, eight PhD students were part of the core team, benefitting from all the training events and collaborative activities. Seven students have already been awarded PhD degrees, with one in the process of writing up her doctoral thesis. Seven students progressed into research or teaching positions in academia and one is working in government policy making. We consider that our programme is an excellent example of the benefit of in-country training and collaborative research that can lead to internationally competitive publications and outputs.

Through the Chem4Energy consortium, we have created a scientific community, which can continue to collaborate scientifically and interact easily via email and Zoom/Teams, including ongoing interaction and collaboration via a continuing annual conference series in southern Africa. The computational aspects of the consortium research and the strong links built with the CHPC in South Africa—which continues to provide the researchers with access to computing resources—is continuing beyond the end of the funded period. Furthermore, KNUST is opening its own materials/molecular modelling research laboratory to postgraduate students from other institutions, and particularly to sub-Saharan African partners. As part of this ongoing plan, KNUST and collaborators have already hosted PhD students from UJ, the Hawassa University in Ethiopia and four exchange students on the International Association for the Exchange of Students for Technical Experience (IAESTE) programme from Germany and India.

The consortium members maintain contact with each other and look for other opportunities for funding UK–African networks. Thus far, engagements with external collaborators or industries have been limited, primarily owing to the disruptions of the COVID-19 restrictions and a shift to online activities. Initially, we had engagements with institutions like NAMPOWER, and the Ministry of Mines and Energy in Namibia, but in 2021, many institutions shifted their priorities to aligning them with online activities. As a result, few activities occurred to build new partnerships and collaborations. However, collaboration with Rossing Uranium Limited (RUL) has been re-ignited, after their initial approach to UNAM to assist in the decontamination of mining tailings. The tailings had accumulated to the extent where these mixtures of different metallic species have started to pose health concerns. The waste mining tailings are believed to contain useful minerals such as manganese, iron, copper and titanium and other valuable metals. Thus, there is significant interest in isolating these metals for further use in research, e.g. as solar energy material production by using the MSc student programme. Materials such as titanium dioxide and copper oxides (obtained from the tailings) can be used in the fabrication of solar materials, through thin films, as well as in catalysis, as was shown in the research by the Chem4Energy ACBI-funded PhD students.

Experience with the Chem4Energy consortium has only strengthened our view of the crucial importance of capacity building and knowledge transfer for the development of excellence in partner institutions. The network of new relationships we have built through our face-to-face consortium meetings and training workshops, and importantly the generous computational resources from CHPC, have enabled competitive research and publications by the PhD students. There has also been a fantastic development in expertise with multi-directional knowledge transfer between all partners, which has fostered collaborations for future projects and which, we trust, will influence the next generation of scientific leaders within Africa and the UK.

## Data Availability

This article has no additional data.

## Dedication

This publication is dedicated to the memory of our valued colleague and friend Professor Richard Tia, one of the investigators from the Kwame Nkrumah University of Science and Techology in Ghana, who sadly died during the project.

## References

[1] Frisch MJ *et al*. 2009 Gaussian 09, Revision B.01. Wallingford, CT: Gaussian, Inc.

[2] Kresse G, Hafner J. 1993 Ab initio molecular dynamics for liquid metals. Phys. Rev. B **47**, 558–561. (10.1103/PhysRevB.47.558) 10004490

[3] Kresse G, Hafner J. 1994 Ab initio molecular-dynamics simulation of the liquid-metal–amorphous-semiconductor transition in germanium . Phys. Rev. B **49**, 14251–14269. (10.1103/PhysRevB.49.14251) 10010505

[4] Kresse G, Furthmüller J. 1996 Efficient iterative schemes for ab initio total-energy calculations using a plane-wave basis set. Phys. Rev. B **54**, 11169–11186. (10.1103/PhysRevB.54.11169) 9984901

[5] Kresse G, Furthmüller J. 1996 Efficiency of ab-initio total energy calculations for metals and semiconductors using a plane-wave basis set. Comput. Mater. Sci. **6**, 15–50. (10.1016/0927-0256(96)00008-0) 9984901

[6] Todorov IT, Smith W, Trachenko K, Dove MT. 2006 DL_POLY_3: new dimensions in molecular dynamics simulations via massive parallelism. J. Mater. Chem. **16**, 1911–1918. (10.1039/b517931a)

[7] Santos-Carballal D, van Sittert C, de Leeuw NH. 2021 Africa-UK partnership for the computer-aided development of sustainable catalysts. S. Afr. J. Chem. (Special Issue) **74**, 1–72. (10.17159/0379-4350/2021/v74a1)

[8] Golovanov N, Lazaroiu GC, Roscia M, Zaninelli D. 2013 Power quality assessment in small scale renewable energy sources supplying distribution systems. Energies **6**, 634–645. (10.3390/en6020634)

[9] Chang CD, Lang WH, Smith RL. 1979 The conversion of methanol and other o‐compounds to hydrocarbons over zeolite catalysts. J. Catal. **47**, 249–259. (10.1002/chin.197918119)

[10] Gogate MR. 2019 New insights into reaction mechanisms of the methanol-to-hydrocarbons (MTH) reactions: the formation of first C–C bond. Pet. Sci. Technol. **37**, 28–37. (10.1080/10916466.2018.1476536)

[11] Ono Y, Mori T. 1981 Mechanism of methanol conversion into hydrocarbons over ZSM-5 zeolite. J. Chem. Soc. Faraday Trans. 1 **77**, 2209. (10.1039/f19817702209)

[12] Humphrey W, Dalke A, Schulten K. 1996 VMD: visual molecular dynamics. J. Mol. Graph. **14**, 33–38, (10.1016/0263-7855(96)00018-5) 8744570

[13] Giannozzi P *et al*. 2009 QUANTUM ESPRESSO: a modular and open-source software project for quantum simulations of materials. J. Phys. Condens. Matter **21**, 395502. (10.1088/0953-8984/21/39/395502) 21832390

[14] Zhao Y, Truhlar DG. 2008 The M06 suite of density functionals for main group thermochemistry, thermochemical kinetics, noncovalent interactions, excited states, and transition elements: two new functionals and systematic testing of four M06 functionals and 12 other functionals. Theor. Chem. Acc. **119**, 215–241. (10.1007/s00214-007-0310-x)

[15] Porter AJ, Botchway CH, Kwakye-Awuah B, Hernandez-Tamargo C, Matam SK, McHugh SL, Silverwood IP, de Leeuw NH, O’Malley AJ. 2022 Local and nanoscale methanol mobility in different H-FER catalysts. Catal. Sci. Technol. **12**, 1663–1677. (10.1039/D1CY02001C)

[16] Botchway CH, Tia R, Adei E, O’Malley AJ, Dzade NY, Hernandez-Tamargo C, de Leeuw NH. 2020 Influence of topology and Brønsted acid site presence on methanol diffusion in zeolites Beta and MFI. Catalysts **10**, 1342. (10.3390/catal10111342)

[17] Botchway CH, Tia R, Adei E, Dzade NY, de Leeuw NH. 2021 H-FER-catalyzed conversion of methanol to ethanol and dimethyl ether: a first-principles DFT study. S. Afr. J. Chem. **74**, 30–35. (10.17159/0379-4350/2021/v74a6)

[18] Nastase SAF, Cnudde P, Vanduyfhuys L, De Wispelaere K, Van Speybroeck V, Catlow CRA, Logsdail AJ. 2020 Mechanistic insight into the framework methylation of H-ZSM-5 for varying methanol loadings and Si/Al ratios using first-principles molecular dynamics simulations. ACS Catal. **10**, 8904–8915. (10.1021/acscatal.0c01454) 32923027 PMC7479850

[19] Roberts VM, Stein V, Reiner T, Lemonidou A, Li X, Lercher JA. 2011 Towards quantitative catalytic lignin depolymerization. Chem. Eur. J. **17**, 5939–5948. (10.1002/chem.201002438) 21472799

[20] Mensah M, Tia R, Adei E, de Leeuw NH. 2022 A DFT mechanistic study on base-catalyzed cleavage of the β-O-4 ether linkage in lignin: implications for selective lignin depolymerization. Front. Chem. **10**, 793759. (10.3389/fchem.2022.793759) 35252111 PMC8892242

[21] Mcdonough TJ. 1993 The chemistry of organosolv delignification. Tappi J. **76**, 186–193.

[22] Agmon N. 1995 The Grotthuss mechanism. Chem. Phys. Lett. **244**, 456–462. (10.1016/0009-2614(95)00905-J)

[23] Chmely SC, Kim S, Ciesielski PN, Jiménez-Osés G, Paton RS, Beckham GT. 2013 Mechanistic study of a Ru-Xantphos catalyst for tandem alcohol dehydrogenation and reductive aryl-ether cleavage. ACS Catal. **3**, 963–974. (10.1021/cs400110r)

[24] Wu A, Patrick BO, Chung E, James BR. 2012 Hydrogenolysis of β-O-4 lignin model dimers by a ruthenium-xantphos catalyst. Dalton Trans. **41**, 11093–11106. (10.1039/c2dt31065a) 22864631

[25] Mensah M, Tia R, Adei E, de Leeuw NH. 2023 C-H versus C-O addition: a DFT study of the catalytic cleavage of the β-O-4 ether linkage in lignin by iridium and cobalt pincer complexes. Catalysts **13**, 757. (10.3390/catal13040757)

[26] Kundu S, Choi J, Wang DY, Choliy Y, Emge TJ, Krogh-Jespersen K, Goldman AS. 2013 Cleavage of ether, ester, and tosylate C(sp3)-O bonds by an iridium complex, initiated by oxidative addition of C-H bonds. experimental and computational studies. J. Am. Chem. Soc. **135**, 5127–5143. (10.1021/ja312464b) 23469859

[27] Nichols JM, Bishop LM, Bergman RG, Ellman JA. 2010 Catalytic C-O bond cleavage of 2-aryloxy-1-arylethanols and its application to the depolymerization of lignin-related polymers. J. Am. Chem. Soc. **132**, 12554–12555. (10.1021/ja106101f) 20731348 PMC2943869

[28] Aghabarari B, Dorostkar N, Martinez-Huerta MV. 2014 Synthesis of biodiesel from Nigella sativa seed oil using surfactant-Brønsted acidic-combined ionic liquid as catalyst. Fuel Process. Technol. **118**, 296–301. (10.1016/j.fuproc.2013.10.003)

[29] Elsheikh YA. 2014 Optimization of novel pyrazolium ionic liquid catalysts for transesterification of bitter apple oil. Process Saf. Environ. Prot. **92**, 828–834. (10.1016/j.psep.2013.05.001)

[30] Freedman B, Pryde EH, Mounts TL. 1984 Variables affecting the yields of fatty esters from transesterified vegetable oils. J. Am. Oil Chem. Soc. **61**, 1638–1643. (10.1007/BF02541649)

[31] Olaoye OE, Oyetunji O, Makhubela BCE, Kumar G, Darkwa J. 2023 Hydrogenation of biodiesel catalysed by pyrazolyl nickel(II) and palladium(II) complexes. RSC Sustainability **1**, 1814–1825. (10.1039/D3SU00254C)

[32] Olaoye OE, Oyetunji O, Makhubela BCE, Muyaneza A, Kumar G, Darkwa J. 2021 Catalytic hydrogenation of sorbic acid using pyrazolyl palladium(II) and nickel(II) complexes as precatalysts. S. Afr. J. Chem. **74**, 50–56. (10.17159/0379-4350/2021/v74a9)

[33] Durndell LJ, Parlett CMA, Hondow NS, Isaacs MA, Wilson K, Lee AF. 2015 Selectivity control in Pt-catalyzed cinnamaldehyde hydrogenation. Sci. Rep. **5**, 9425. (10.1038/srep09425) 25800551 PMC4371104

[34] Nemamcha A, Rehspringer JL, Khatmi D. 2006 Synthesis of palladium nanoparticles by sonochemical reduction of palladium(II) nitrate in aqueous solution. J. Phys. Chem. B **110**, 383–387. (10.1021/jp0535801) 16471546

[35] Xu L, Wu XC, Zhu JJ. 2008 Green preparation and catalytic application of Pd nanoparticles. Nanotechnology **19**, 305603. (10.1088/0957-4484/19/30/305603) 21828765

[36] Lara LRS, Zottis AD, Elias WC, Faggion D, Maduro de Campos CE, Acuña JJS, Domingos JB. 2015 The catalytic evaluation of in situ grown Pd nanoparticles on the surface of Fe_3_O_4_@dextran particles in the p-nitrophenol reduction reaction . RSC Adv. **5**, 8289–8296. (10.1039/C4RA16440G)

[37] Pelagatti P. 2006 Palladium and platinum. In The handbook of homogeneous hydrogenation. Hoboken, NJ: Wiley. (10.1002/9783527619382.ch4)

[38] Sejie FP, Oyetunji OA, Darkwa J, Beas IN, Makhubela BCE, Dzade NY, de Leeuw NH. 2023 The transfer hydrogenation of cinnamaldehyde using homogeneous cobalt(II) and nickel(II) (E)-1-(pyridin-2-yl)-N-(3-(triethoxysilyl)propyl)methanimine and the complexes anchored on Fe3O4 support as pre-catalysts: an experimental and in silico approach. Molecules **28**, 659. (10.3390/molecules28020659) 36677718 PMC9865650

[39] Nyepetsi M, Oyetunji OA, Mbaiwa F. 2024 ReaxFF study of the decarboxylation of methyl palmitate over binary metallic nickel-molybdenum catalysts. Mol. Simul. **50**, 1–13. (10.1080/08927022.2023.2283539)

[40] Ma F, Clements LD, Hanna MA. 1999 The effect of mixing on transesterification of beef tallow. Bioresour. Technol. **69**, 289–293. (10.1016/S0960-8524(98)00184-9)

[41] Ochoa-Hernández C, Yang Y, Pizarro P, de la Peña O’Shea VA, Coronado JM, Serrano DP. 2013 Hydrocarbons production through hydrotreating of methyl esters over Ni and Co supported on SBA-15 and Al-SBA-15. Catal. Today **210**, 81–88. (10.1016/j.cattod.2012.12.002)

[42] Bie Y, Gutierrez A, Viljava TR, Kanervo JM, Lehtonen J. 2013 Hydrodeoxygenation of methyl heptanoate over noble metal catalysts: catalyst screening and reaction network. Ind. Eng. Chem. Res **52**, 11544–11551. (10.1021/ie4012485)

[43] Sankar M *et al*. 2012 Synthesis of stable ligand-free gold-palladium nanoparticles using a simple excess anion method. ACS Nano **6**, 6600–6613. (10.1021/nn302299e) 22769042

[44] Amakali T *et al*. 2022 Photocatalytic degradation of rhodamine B dye and hydrogen evolution by hydrothermally synthesized NaBH_4_-spiked ZnS nanostructures. Front. Chem. **10**, 835832. (10.3389/fchem.2022.835832) 35494625 PMC9046778

[45] Amakali T, Daniel LS, Uahengo V, Dzade NY, de Leeuw NH. 2020 Structural and optical properties of ZnO thin films prepared by molecular precursor and sol–gel methods. Crystals (Basel) **10**, 132. (10.3390/cryst10020132)

[46] Živković A, Farkaš B, Uahengo V, de Leeuw NH, Dzade NY. 2019 First-principles DFT insights into the structural, elastic, and optoelectronic properties of α and β-ZnP_2_: implications for photovoltaic applications. J. Phys. Condens. Matter **31**, 265501. (10.1088/1361-648X/ab111c) 30889559

[47] Farkaš B, Živković A, Uahengo V, Dzade NY, de Leeuw NH. 2022 First-principles DFT insights into the stabilization of zinc diphosphide (ZnP_2_) nanocrystals via surface functionalization by 4-aminothiophenol for photovoltaic applications. ACS Appl. Energy Mater. **5**, 2318–2328. (10.1021/acsaem.1c03804)

[48] Farkaš B, Živković A, Uahengo V, Dzade NY, de Leeuw NH. 2021 Insights from density functional theory calculations into the effects of the adsorption and dissociation of water on the surface properties of zinc diphosphide (ZnP_2_) nanocrystals. Phys. Chem. Chem. Phys. **23**, 26482–26493. (10.1039/d1cp02784k) 34806732

[49] Živković A *et al*. 2021 Structural and electronic properties of Cu₄O₃ (paramelaconite): the role of native impurities. Pure Appl. Chem. **93**, 1229–1244. (10.1515/pac-2021-0114)

